# The dual role of whole-genome duplication: biological mechanisms, functional consequences, and detection advances: The dual role of whole-genome duplication and its detection

**DOI:** 10.3724/abbs.2025175

**Published:** 2025-09-30

**Authors:** Yawei Song, Jiajie Yang, Shuheng Wu, Wei Wu

**Affiliations:** Key Laboratory of Multi-Cell Systems Shanghai Institute of Biochemistry and Cell Biology Center for Excellence in Molecular Cell Science Chinese Academy of Sciences University of Chinese Academy of Sciences Shanghai 200031 China

**Keywords:** whole-genome duplication, polyploidy, aneuploidy, cell division, chromosome instability

## Abstract

Whole-genome duplication (WGD) represents an evolutionarily conserved process occurring in prokaryotes, eukaryotes, and somatic mammalian tissues. While developmentally programmed WGD supports normal tissue regeneration, unscheduled WGD drives chromosomal instability and oncogenic progression in cancer. Recent studies have clarified dual roles of WGD across physiological homeostasis and disease pathogenesis. Here, we review the prevalence of WGD, the molecular mechanisms driving its major causes and its biological consequences. In addition, we highlight recent advancements in WGD detection, including both conventional cytogenetic techniques and newly developed high-throughput sequencing approaches. The integration of multi-omics and machine learning further improves ploidy analysis, particularly in cancer research. Together, these insights establish WGD as a critical regulator of development, regeneration, and disease and underscore the importance of emerging computational and sequencing tools for its precise characterization.

## Introduction

Whole-genome duplication (WGD), which is defined as the duplication of the entire set of chromosomes within a cell, represents the largest known genetic mutation event [1]. Although WGD was initially considered a rare event, advances in technology and its widespread application have revealed WGD to be a pervasive biological phenomenon. It occurs across a broad range of organisms, tissues and pathological conditions, providing raw genetic material for evolutionary innovation and serving as a key driver of genetic diversity, species divergence, and malignant transformation [2]. In this review, we summarize the prevalence of WGD at both the organismal and cellular levels and then describe the four principal mechanisms leading to WGD (cell fusion, endoreplication, mitotic slippage, and cytokinesis failure). Following this, we discuss the biological consequences of WGD and present an overview of current technologies for detecting cellular ploidy. Given the breadth of topics covered, an exhaustive dissection of molecular mechanisms is beyond the scope of this review, and we recommend other specialized mechanistic reviews for further details [ 3– 5] .

## Prevalence of Whole-Genome Duplication

### Polyploid organisms

In prokaryotes, evidence of polyploidy was first reported in studies on *Escherichia coli*, and subsequently, it has been described in many other prokaryotic lineages. Many bacteria and archaea maintain multiple copies of individual chromosomes (polyploid), particularly among the majority of clades of Euryarchaeota (e.g., Halobacteriales, Methanosarcinales, Thermococcales, and Methanococcales), where polyploidy appears to be a defining characteristic [ 6– 8] . Additional chromosomal copies can provide intact templates for DNA repair under stressful conditions, such as ionizing radiation or oxidative stress, and can also restrain the phenotypic expression of deleterious recessive mutations [9].

Although fungal life cycles are often dominated by the haploid state, similar to most prokaryotes, advances in whole-genome sequencing (WGS) have revealed a growing prevalence of polyploidy across fungal species [10]. For instance, the prevalent opportunistic pathogen *Candida albicans*, which has long been considered an asexual, highly heterozygous, obligate diploid organism, has been found to harbor tetraploid strains in both laboratory and natural environments [ 11, 12] . Another model organism, *Saccharomyces cerevisiae* (unicellular baker′s yeast), can undergo asexual reproduction in stable haploid, diploid, and polyploid forms, making it an important model for studying ploidy [13]. Furthermore, in an *in vitro* evolution experiment, haploid *S*. *cerevisiae* frequently underwent diploidization under glucose-limited conditions, indicating that WGD events play a significant biological role in its environmental adaptation [ 14, 15] .

Polyploidy is widespread in plants, and the proportion of polyploid species increases progressively from the equator to the poles: in tropical regions, the proportion of polyploid species is less than 20%; in temperate zones, the proportion accounts for 38–40%; in taiga ecosystems, the proportion increases to 47%; and in tundra ecosystems, the proportion reaches approximately 51% [16]. These findings suggest that polyploidy, which provides genetic diversity and functional gene redundancy through genome duplication, enhances environmental adaptability and stress tolerance [17]. For example, naturally occurring tetraploid *A*. *thaliana* accessions exhibit increased salt tolerance via the regulation of leaf potassium levels, further supporting this phenomenon [18].

Traditional perspectives often mistakenly assumed that polyploidy does not occur in animals. However, an increasing body of evidence has demonstrated its presence in many animal species. Among invertebrates, polyploidy is particularly common in the annelid subclass Oligochaeta, where the proportion of polyploid and diploid individuals in earthworms is nearly equal [19]. In vertebrates, genome evolution initially underwent two rounds of ancient WGD events, which helped shape the structure of modern vertebrate genomes [20]. A third WGD occurred in the ancestor of all teleost fishes, an evolutionary event known as teleost-specific genome duplication (TGD), which doubled all chromosomes and genes. Although most duplicated genes have reverted to single copies over time, a substantial proportion have retained two copies, called ohnologs. These ohnologs have left a lasting imprint on the genomes of extant teleosts, and WGD events are believed to have played a critical role in making teleosts the most species-rich clade among vertebrates [ 21, 22] . Beyond these ancient events, some teleost lineages have experienced additional rounds of WGD. For instance, polyploidization occurred independently multiple times within Cyprinidae. One study revealed at least 13 independent WGD events in Cyprinidae, with approximately 400 of the more than 1300 species being polyploid (although it remains possible that polyploidy is still uncharacterized in some species) [23]. Among amphibians, polyploidy is also highly prevalent, particularly in African clawed frogs ( *Xenopus*). Among the 27 extant species in this genus, only one is not polyploidy [24].

In contrast, polyploidy is extremely rare in reptiles and typically manifests as triploids. These triploid individuals often reproduce via parthenogenesis [25]. While spontaneously generated, viable triploids have been reported in birds, they are invariably sterile and thus cannot give rise to new species. Whole-organism polyploidization is extraordinarily rare in mammals, as it generally results in embryonic resorption or spontaneous abortion [ 26, 27] . There have been claims that the red viscacha rat (Tympanoctomys barrerae) and its close relatives are fully tetraploid [28], but subsequent research suggests that their large genome size is more likely due to the amplification of diverse repetitive sequences rather than true WGD [29].

### Programmed whole-genome duplication in tissues

In early evolution, ancient WGD events, such as teleost-specific polyploidization, not only drove morphological and physiological diversification but also provided models for investigating how extant species gain adaptive advantages through polyploidization. Notably, polyploid organisms typically exhibit improved stress resistance and increased adaptability to environmental fluctuations [30]. Although rare in mammals, many tissues still retain highly programmed WGD mechanisms throughout embryonic development and individual growth. These tightly regulated genome duplication events enable various tissues to address microenvironmental changes, the developmental, functional, and stress-response demands of various tissues. Thus, in addition to exploring polyploid organismal diversity and its role in environmental adaptation, we also investigated tissue-specific WGD in mammals and its functional significance. These localized polyploidization events enable tissue- and organ-level stress adaptation, highlighting the key role of WGD in biological function and adaptive responses [3].

In some animals, polyploid cells arise during the embryonic stage and are found primarily in embryonic supporting structures, where they play critical roles in barrier formation and structural support [31]. For instance, despite significant morphological differences between the placentas of mice and humans, both exhibit polyploid features. Placental syncytiotrophoblasts and trophoblast giant cells contain multiple copies of the genome. This polyploidization not only increases cell size and metabolic activity but also supports the core functions of the placenta, such as nutrient transport, gas exchange, and hormone secretion, thereby ensuring proper embryonic development [ 32, 33] .

WGD events also occur within the immune system. Granulomas, which form in response to persistent inflammatory stimuli, are tightly organized aggregates of immune cells that exhibit features of polyploidization [34]. Additionally, polyploidization is a prominent feature of mature osteoclasts, which are specialized bone-resorbing cells derived from myeloid precursors in the hematopoietic system. Compared with their diploid counterparts, polyploid osteoclasts exhibit significantly greater resorptive efficiency, contributing critically to bone remodeling [ 35, 36] .

In the circulatory system, up to 70% of human and 85% of rodent cardiac myocytes are polyploid [37]. Most ventricular cardiomyocytes in adult mammals exhibit a tetraploid state, and the proportion of cardiomyocytes undergoing WGD is significantly greater in the left ventricle than in the right ventricle [38]. These findings suggest that larger, polyploid cardiomyocytes may be better adapted to the greater mechanical load experienced by the continuously contracting myocardial tissue. Moreover, in response to cardiomyocyte loss or injury caused by cardiac stress, such as myocardial infarction, and owing to the limited regenerative capacity of the postnatal mammalian heart, cardiomyocytes often compensate by reinitiating DNA synthesis, leading to further polyploidization [39].

The liver is one of the few mammalian organs that exhibit dynamic polyploidy during normal homeostasis, regeneration and in response to injury, and its WGD phenomenon has been extensively studied. Polyploid cells in the liver parenchyma were first discovered more than a century ago and are considered physiological adaptations during development [40]. In mice, hepatocyte polyploidization begins within the first two weeks after birth and persists throughout adulthood. This process primarily occurs through cytokinesis failure: diploid hepatocytes undergo nuclear division but fail to complete cytokinesis, resulting in the formation of binucleated tetraploid hepatocytes [40]. A similar phenomenon is observed in the human liver, although at a lower frequency. In human livers, approximately 50% of hepatocytes are polyploid, whereas in adult rodents [ 41, 42] , this proportion reaches as high as 90% [ 43, 44] . In these species, the number of polyploid hepatocytes increases with age, providing protection against genotoxic damage [45]. The liver is also known for its remarkable regenerative capacity. In the adult human liver parenchyma, hepatocytes are replaced at a slow rate, with a mean lifespan of approximately 200–300 days. Under stress conditions such as oxidative stress, telomere damage, and iron overload, polyploidy can be induced to increase metabolic capacity, protect against DNA damage and provide genetic variability through multiple gene copies [45]. Partial hepatectomy-induced liver regeneration serves as a classic model of compensatory hypertrophy. During this regenerative process, the dynamics of hepatocyte polyploidization shift significantly. In rats subjected to partial hepatectomy, all hepatocytes re-enter the cell cycle, and by the end of the regeneration process, the number of binucleated polyploid hepatocytes decreases, whereas the population of mononucleated polyploid hepatocytes increases [46]. Only when a substantial portion of the liver is resected does proliferation significantly contribute to regeneration [47].

Skeletal muscle, the largest organ in the human body (accounting for approximately 40% of body weight), shows a high degree of plasticity, with its mass being subject to changes caused by factors such as physical exercise, aging, injury, and pathological conditions. Throughout evolution, multinucleation and polyploidization have been hallmarks of muscle fibers and play critical roles in coordinating muscle contraction and movement [ 48, 49] . Localized physical or chemical trauma can induce rapid focal necrosis of muscle fibers, during which a newly formed membrane segregates the surviving ends from the necrotic region and triggers a localized immune response [50]. In response to signals derived from the damaged area, satellite cells begin to proliferate, differentiate, and fuse with one another or with existing muscle fibers, once again undergoing polyploidization [51].

In animals, there are some tissues where polyploidization, although less associated with developmental processes, is a mechanism for tissue repair or regeneration. Post-injury epithelial polyploidization and the resulting hypertrophy have been observed across species ranging from flies to vertebrates and occur in various tissue systems [52]. The urothelium, an epithelial barrier lining the lumen of the urinary tract, consists of binucleated, octoploid superficial cells that resist pathogens and toxins. In the adult urothelium, which is otherwise quiescent, acute injury caused by urinary tract infection (UTI) or exposure to toxins can trigger rapid regeneration via polyploidization, thereby maintaining the integrity of the urothelial barrier [53]. Similarly, lung injury during the acute damage response can lead to hypertrophy and polyploidization of alveolar type 2 (AT2) cells, particularly through the formation of binucleated AT2 cells [54].

This programmed polyploidization serves as a highly conserved and adaptive mechanism to restore tissue integrity across diverse physiological contexts. However, polyploidization is not always a regulated process, and aberrant polyploidy has been increasingly recognized in pathological states, particularly in cancer.

### Unscheduled whole-genome duplication in cancer

Controlled polyploidization has essential biological functions in various normal cell types, including hepatocytes, megakaryocytes, cardiomyocytes and trophoblasts [3]. However, WGD can also occur unscheduled in proliferative tissues, where it may act as a driver of tumorigenesis by increasing chromosomal instability (CIN), thereby increasing tumor heterogeneity, aggressiveness and therapy resistance [55].

The oncogenic potential of tetraploid cells was first hypothesized by Theodor Boveri more than a century ago and has been experimentally validated [56]. For example, tetraploid p53 ^-/-^ mouse mammary epithelial cells, induced by cytokinesis failure via dihydrocytochalasin B (DCB), formed malignant tumors in nude mice, whereas their matched diploid cells did not [57]. Furthermore, multiple studies have confirmed that some tumors undergo WGD at early stages [58] or show significant increases in WGD-positive cells during progression [ 59, 60] , supporting the notion that WGD is a common event in cancer evolution and plays a pivotal role in tumor initiation and progression.

Loss of p53 function is a major permissive factor for WGD. It enables tetraploid cells to bypass cell cycle checkpoints, evade apoptosis, and re-enter the replication cycle. Indeed, WGD occurs more frequently in tumors harboring TP53 mutations than in TP53 wild-type tumors. In temporally resolved tumor samples, ~97.3% of WGD events occurred after TP53 inactivation [61], indicating that p53 loss is a common prerequisite. However, p53 deficiency is not strictly needed. Approximately half of WGD events occur in TP53-intact tumors and are often accompanied by aberrant activation of cell cycle drivers, particularly the E2F pathway. The amplification of Cyclin E1 (CCNE1) has been strongly associated with WGD. The overexpression of Cyclin E induces replication stress, unscheduled origin firing, and DNA damage accumulation, ultimately triggering WGD in the absence of proper mitotic entry [62]. This mechanism is observed in multiple tumor types and is particularly prominent in non-small cell lung cancer (NSCLC), which has one of the highest WGD frequencies. A recent integrative proteogenomic analysis revealed that these subtypes not only display WGD-associated features but also harbor amplifications of cell cycle regulators such as SOX2 and TP53, as well as hyperactivation of signaling pathways, including PI3K-Akt and cyclin-dependent kinases (CDK) (CDK1, CDK2, and CDK4), reinforcing the role of cell cycle dysregulation in WGD induction [63].

Beyond the tetraploidy checkpoint, the spindle assembly checkpoint (SAC) is an essential mechanism that ensures proper chromosomal segregation. SAC delays anaphase onset until all kinetochores are properly attached to bipolar spindles, thus preventing chromosome missegregation [64]. However, SAC integrity is often compromised following WGD, particularly in tetraploid cells, leading to increased mitotic errors and micronuclei formation. SAC dysfunction exacerbates CIN and is considered a key driver of malignant transformation in WGD cells [58]. Notably, SAC impairment is frequently observed in early-stage tetraploid or aneuploid tumors, suggesting a role in early tumorigenesis and potential utility as a prognostic biomarker.

The impact of WGD on tumorigenesis is highly context dependent and influenced by genetic background, physiological state, and microenvironmental factors. A key consequence of WGD is a global increase in genomic content, which confers genetic redundancy that buffers deleterious mutations and enhances cellular adaptability to stress and therapy. Moreover, WGD accelerates clonal evolution, increases intratumoral heterogeneity, and promotes treatment resistance. It also drives large-scale chromosomal rearrangements and contributes to aneuploidy. For example, in pancreatic cancer, chromothripsis events were predominantly observed on chromosomes 8 and 15 prior to WGD, whereas post-WGD rearrangements affected chromosomes 13, 16, and 18, correlating with a worse prognosis [65]. These findings highlight WGD as a molecular tipping point from non-invasive to aggressive disease states. WGD-derived tetraploid cells are inherently unstable and prone to mitotic errors. The resulting micronuclei can rupture, releasing genomic DNA into the cytoplasm and activating the cGAS–STING pathway along with non-canonical NF-κB signaling. This inflammatory cascade promotes tumor invasion and metastasis, whereas suppression of this axis can delay metastatic progression [66]. In addition, WGD has led to profound remodeling of the 3D genome. For example, oncogenes can be repositioned into transcriptionally active A compartments in mononucleated, p53-deficient WGD cells, whereas tumor suppressors can shift into repressive B compartments, thereby facilitating malignant transformation [67].

Despite the adaptive advantages conferred by WGD, it also creates unique vulnerabilities. WGD cells exhibit reduced replication origin efficiency and accumulate extensive DNA damage during the first S phase, primarily due to insufficient replication machinery [68]. To mitigate this damage, cells activate tumor suppressor pathways such as the p53 and Hippo pathways to restrict proliferation [69]. Furthermore, WGD-positive tumor cells are highly dependent on specific mitotic regulators. For example, depletion of KIF18A, a spindle-associated kinesin, selectively induces lethality in WGD cells while sparing diploid cells, highlighting a synthetic lethal interaction [ 58, 70] . These findings nominate KIF18A and similar factors as promising therapeutic targets in WGD-driven cancers and point toward new avenues for precision oncology.

## Molecular Mechanisms of Whole-Genome Duplication

**Figure 1:**
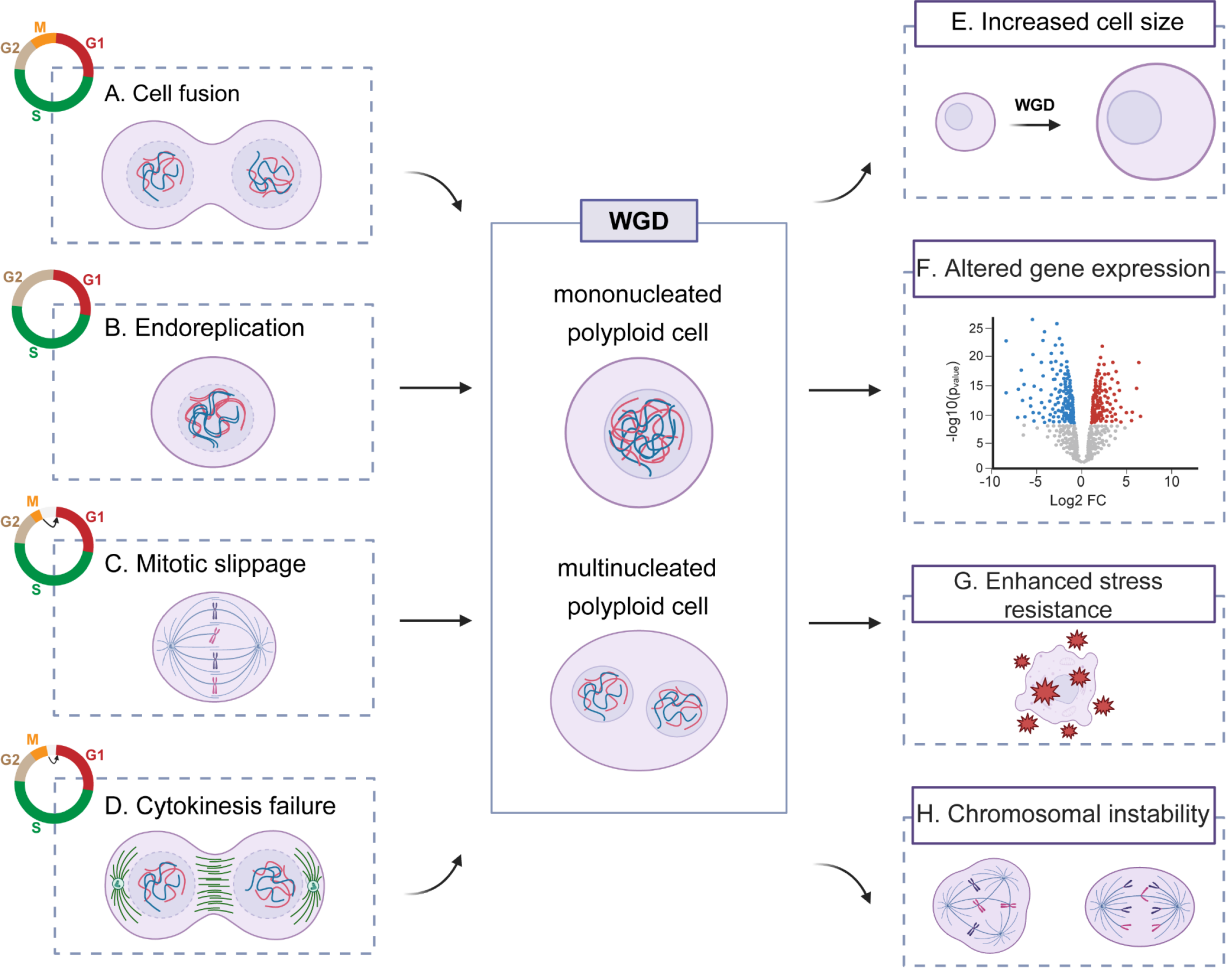
Figure 1 Mechanisms of WGD and its biological consequences (A–D) are four representative mechanisms of WGD induction. (A) Cell fusion: Fusion between cells or with themselves results in the combination of nuclei and cytoplasm, producing multinucleated or polyploid cells. (B) Endoreplication: Cells bypass mitosis and undergo repeated rounds of DNA replication, leading to mononucleated polyploid cells. (C) Mitotic slippage: Cells enter mitosis but fail to complete it due to prolonged spindle assembly checkpoint activation, leading to exit from mitosis without chromosome segregation or cytokinesis, forming polyploid cells. (D) Cytokinesis failure: Disruption of cleavage furrow ingression or abscission causes failure of cell division, resulting in binucleated tetraploid cells. Color coding of cell cycle phases is shown in circular diagrams: red = G1, green = S, neutral beige = G2, orange = M. (E–H) are main biological consequences of WGD. (E) Increasing with size: WGD typically leads to increased cell and nuclear size, a feature observed in both physiological and pathological contexts. (F) Effects on gene expression: While global gene expression remains largely stable post-WGD, polyploid cells often exhibit upregulated expression of tissue-specific functional genes, enhancing specialized cellular roles. (G) Enhance stress resistance: Polyploidy confers resilience by buffering genetic damage and upregulating stress response pathways, contributing to tissue protection and repair. (H) Chromosomal instability: Despite its benefits, WGD can disrupt chromosome segregation, especially in the absence of p53, increasing the risk of aneuploidy and genomic instability, thereby promoting tumorigenesis. Central box: These mechanisms contribute to the formation of mononucleated or multinucleated polyploid cells, representing different outcomes of WGD. The figure was created by Biorender.

WGD occurs widely across organisms, tissues, and pathological conditions, holding significant biological importance. However, through which mechanisms do cells bypass the strict diploid state? This process involves the following four main mechanisms ( Figure 1): (1) Cell fusion: two or more cell membranes merge to form a single cell, thus doubling the DNA context; (2) endoreplication: a process in which cells replicate their DNA repeatedly without undergoing cell division (consisting only of G and S phases) [71]; (3) mitotic slippage: improperly attached or unattached centromeres can delay mitotic exit and ultimately lead to slippage from mitosis [72]; and (4) cytokinesis failure: during the final stage of cell division, cytoplasmic division is not successfully completed. Endoreplication, mitotic slippage, and cytokinesis failure occur at distinct stages of the cell cycle. In contrast, cell fusion can take place in any cell cycle phase, although it most commonly occurs during interphase (the non-mitotic stage), especially before the cell enters mitosis [73].

### Cell fusion

Cell fusion refers to the process by which cells merge with each other or with themselves, and it is involved in many physiological and pathophysiological processes ( Figure 1A) [74]. Generally, cell fusion occurs in three distinct stages: (1) contact between two membrane compartments; (2) hemifusion; and (3) opening and expansion of the fusion pore. During the initiation phase, the lipid bilayers approach each other, followed by membrane‒membrane interactions. The distance between the two membranes is approximately 10–20 nanometers, and the membrane is filled with membrane-associated proteins, such as viral envelope proteins. The membrane surfaces are also covered by a dense network of proteins [75]. These proteins form protein-depleted patches within opposing membranes, preventing the initiation of cell fusion. For example, interactions between the cytoskeleton and membrane proteins can impede protein mobility, and fusion occurs only after such barriers are removed.

Certain types of cells possess the ability to form syncytia or polyploid cells, which are essential for species propagation, development, and the maintenance of normal physiological functions. Sperm-oocyte fusion during fertilization represents the earliest and most well-known example of cell fusion [76]. In gamete fusion, the binding of sperm to oocytes is mediated by IZUMO1 on the sperm surface and CD9 on the microvilli of the oocyte. IZUMO1 interacts with the receptor JUNO to form an adhesion complex that enables specific recognition between sperm and oocytes, but the precise mechanism of plasma membrane fusion remains unclear and may involve other unidentified fusogens [ 77– 79] . In contrast, myoblast fusion requires prior cellular differentiation, accompanied by molecular events such as adhesion, migration, and cytoskeletal rearrangement. The recently discovered fusogen Myomaker plays a critical role in this process, as it not only regulates the formation of muscle fibers but also induces non-fusogenic cells to become multinucleated [ 80, 81] . These mechanisms highlight the specificity and diversity of cell fusion processes, which are important for normal development and physiological functions in multicellular organisms.

Cell fusion is also an important mechanism for WGD formation. Multiple pathways can promote tumor cell fusion, contributing to cancer development, metastasis, and other malignant processes. Enveloped viruses may trigger cell fusion by infecting tumor cells, a phenomenon particularly evident in infection-associated cancers such as liver and cervical cancer [82]. Virus-induced cell fusion, especially when combined with p53 inactivation or oncogene deregulation, can lead to genome doubling or karyotypic randomization in diploid human fibroblasts, resulting in extensive CIN [83]. Additionally, the abnormal expression of physiological fusogens in tumor cells can drive fusion. For example, the Env protein of the human endogenous retrovirus HERV-W, which mediates fusion in trophoblast cells, has also been found to mediate the fusion of breast cancer cells with endothelial cells [82]. Moreover, cell fusion may occur as a byproduct of “cell cannibalism”, such as the phagocytosis of tumor cells by macrophages or endocytosis between tumor cells [ 84– 86] .

### Endoreplication

Endoreplication (also referred to as endoreduplication) is a modified cell cycle in which the nuclear genome undergoes replication without subsequent mitosis, leading to increased DNA content and polyploidy ( Figure 1B). In the endoreplication cycle (also known as the endocycle), cells transition directly from the G2 to the G1 phase following a round of DNA synthesis (S phase), bypassing key mitotic events such as nuclear envelope breakdown and chromosomal condensation [87].

The molecular mechanisms underlying endoreplication involve multiple layers of cell cycle regulatory networks. One evolutionarily conserved mechanism is the suppression of mitosis-associated CDK activity via ubiquitin-mediated proteolysis, thereby blocking mitotic entry and promoting the initiation of endoreplication [88]. In the canonical cell cycle, the Cyclin E/Cdk2 complex initiates S phase, Cyclin A/Cdk2 sustains S phase progression, and Cyclin B/Cdk1 drives entry into mitosis [89]. Therefore, the induction of endoreplication requires the inhibition of these Cyclin/CDK complexes, particularly the suppression of Cyclin B/Cdk1 activity. The anaphase-promoting complex/cyclosome (APC/C) plays a pivotal role in this process: its substrate recognition subunit Fzr/Cdh1 (known as Rap in *Drosophila*) regulates APC/C to degrade Cyclin A and Cyclin B, maintaining low Cdk1 activity to prevent mitotic entry [ 89, 90] . This mechanism is considered a key driver of the endoreplication program. In addition, cyclin-dependent kinase inhibitors (CKIs) are critical in the regulation of endoreplication [91]. For instance, mammalian p21 and p57 can directly inhibit the kinase activity of Cyclin/CDK complexes, facilitating mitotic exit and promoting entry into the endoreplicative cycle [ 92– 94] .

Endoreplication plays critical roles in the development of various tissues across diverse organisms and is often associated with terminal differentiation, typically resulting in irreversible characteristics [71]. For example, in Drosophila salivary gland cells, endoreplication depends on the periodic oscillation of the transcription factor E2F1. E2F1 accumulates during the G phase and activates the CycE/Cdk2 complex to trigger S phase entry, after which it is degraded by CRL4 ^CDT2^, promoting the formation of giant polytene chromosomes [ 95, 96] . In mammals, CKIs similarly regulate differentiation-associated endoreplication. For instance, p57 is essential for suppressing Cdk1 activity during the differentiation of trophoblast stem cells into trophoblast giant cells (TGCs), ensuring mitotic exit and polyploidization [94]. Endoreplication also plays an essential role in tissue regeneration and damage repair. For instance, the insulin signaling pathway (IIS) regulates endoreplication in Drosophila follicular epithelium through mechanotransduction [97], whereas the EGFR/RAS/MAPK pathway functions independently of IIS to regulate intestinal epithelium regeneration in Drosophila [98]. Despite tissue-specific differences in signaling inputs, these processes rely on precise coordination between cell cycle regulators and signaling pathways to maintain tissue function and integrity.

Moreover, endoreplication has complex implications in cancer, where genome instability arising from polyploidy is a recognized risk factor. Clinically, endoreplication and polyploidy have been observed in various cancers, with incidence rates ranging from 11% in gastric cancer to 54% in hepatic adenocarcinoma [ 56, 71] . In human hepatocytes, dysregulation of the Hippo-Yap signaling pathway induces mitotic arrest and polyploidy via Akt-mediated acetylation of Skp2, leading to the accumulation of the CDK inhibitor p27, which is associated with the development of hepatocellular carcinoma [99].

### Mitotic slippage (endomitosis)

The term endomitosis was originally used to describe a rare cell cycle in which mitosis takes place without nuclear envelope breakdown or cytokinesis ( Figure 1C) [100]. Currently, however, endomitosis (or mitotic slippage) refers to the process in which cells complete DNA replication but fail to finish mitosis, resulting in mononucleated or binucleated polyploid cells [71]. The outcome depends on whether the M phase is aborted before or after the initiation of sister chromatid segregation, which normally occurs during anaphase [101].

SAC, the key regulator of mitotic fidelity, delays anaphase onset by inhibiting APC/C via the diffusible mitotic checkpoint complex (consisting of MAD2, BUBR1 and CDC20) [89]. This surveillance mechanism ensures accurate chromosome alignment prior to segregation. Nevertheless, even in the presence of an active SAC, Cyclin B is gradually degraded through low-level APC/C activity, leading to a progressive decline in Cdk1 activity and eventual mitotic exit without chromosome segregation, defined as mitotic slippage [89].

Incomplete nuclear division during endomitosis is a characteristic feature of mammalian megakaryocytes [102]. During endomitosis in megakaryocytes, nuclear envelope breakdown, chromosomal condensation, and multipolar spindle formation are observed. However, after chromatid separation, anaphase B and cytokinesis fail to occur. Instead, the nuclear envelope reforms during a telophase-like stage, and cytokinesis is entirely bypassed. In response to thrombopoietin, Cyclin D3 is upregulated to promote polyploidization up to 128N, thereby supporting extensive platelet production [103].

In cancer cells, mitotic slippage represents a mechanism by which cells evade death and is considered a major contributor to resistance against microtubule-targeting chemotherapeutic agents [ 104, 105] . Mitotic slippage occurs during mitotic arrest, where the rapid degradation of Cyclin B1, in conjunction with the slow induction of pro-apoptotic signals (or the slow degradation of pro-survival proteins), collectively contributes to this phenomenon [106]. In acute myeloid leukemia (AML), studies using oxindole-1 (OX-1) have demonstrated a direct relationship between Cyclin B1 degradation and mitotic slippage [107]. Specifically, OX-1 treatment promotes the gradual degradation of Cyclin B1, thereby impairing the apoptotic function of the maturation-promoting factor (MPF) complex and ultimately facilitating mitotic slippage, which leads to the generation of viable polyploid cells [108].

### Cytokinesis failure

Cytokinesis, the final stage of mitosis, is essential for the accurate segregation of duplicated genetic material and organelles into two daughter cells ( Figure 1D). This process comprises a series of tightly regulated, temporally coordinated steps, including cleavage plane specification, furrow ingression, midbody formation, and abscission. Each step is highly interdependent, with the successful completion of one phase required for the initiation of the next [109].

Central spindle formation is critical for the completion of mitosis and is regulated by the chromosomal passenger complex (CPC), which consists of Aurora B kinase, INCENP, Survivin, and Borealin. This process also requires the coordinated function of PRC1, KIF4, and Mklp1. Aurora B promotes the recruitment of Mklp1, a component of the centralspindlin complex, to the spindle midzone by phosphorylating it at Ser708. In parallel, PRC1 and KIF4 cooperate to stabilize and assemble the central spindle [110]. In the equatorial cortex, the CPC and centralspindlin complex jointly regulate the localized activation of RhoA, which directs the assembly and constriction of the actomyosin contractile ring. RhoA activation is dependent on its upstream guanine nucleotide exchange factor, Ect2 (a RhoGEF), whose localization and activity are regulated by Cyk4 [111]. As the contractile ring constricts, the plasma membrane progressively ingresses, leading to the formation of a midbody, which serves as a platform for abscission. The final membrane scission event is mediated by the ESCRT-III (endosomal sorting complex required for transport III) machinery, which localizes to the intercellular bridge and executes membrane fission through polymerization and mechanical constriction [112]. The timing of abscission is dynamically regulated by Plk1 and Aurora B kinases. Notably, in the presence of chromatin bridges, sustained Aurora B activity delays abscission to prevent chromosome breakage [113].

As a highly orchestrated, multi-step process, cytokinesis is vulnerable to perturbations at any stage, which can result in failure. In hepatocytes, the primary mechanism underlying polyploidization is cytokinesis failure. Structural defects in the actin cytoskeleton are potentially important causes of this failure. These defects can impair the interaction between astral microtubules and the cell cortex, leading to disrupted signal transduction, which in turn affects RhoA activation and cleavage furrow ingression. As a result, cytokinesis becomes defective, ultimately leading to the formation of binucleated tetraploid hepatocytes [ 44, 114] .

Cytokinesis failure is a major mechanism underlying the generation of tetraploid cells and may play a critical role in tumorigenesis. Tetraploid cells are highly prone to chromosome missegregation during subsequent cell divisions, which can lead to the formation of aneuploid cells and micronuclei [115]. Moreover, when cytokinesis occurs in the presence of lagging chromosomes, it can induce DNA double-strand breaks and trigger p53-dependent G1 cell cycle arrest. In the absence of functional p53, these structural abnormalities are more likely to accumulate, thereby promoting tumor initiation and progression [ 116, 117] .

## Consequences of Whole-Genome Duplication

WGD is not merely a genomic event; it triggers a cascade of profound changes at the cytological, molecular, biological, and physiological levels, thereby reshaping the biological characteristics of affected cells. Whether arising from programmed physiological processes or unscheduled events such as tumorigenesis or cellular stress, WGD has had both distinct and overlapping biological consequences that profoundly influence cell fate, function, and evolutionary potential.

### Direct impact on cell size

One of the most immediate consequences of WGD is an increase in cell size ( Figure 1E). In general, there is a positive correlation between cell size and DNA ploidy, a phenomenon widely observed across various eukaryotic organisms, from yeast to mice [ 118, 119] . During normal development, organisms leverage the increased cell size conferred by WGD to generate large, specialized cells that fulfill tissue-specific structural and functional demands. For example, Drosophila larval salivary gland cells, mammalian skeletal muscle fibers, megakaryocytes, and trophoblast giant cells acquire increased cell size and enhanced functionality through programmed WGD [87].

In contrast, abnormal increases in cell and nuclear size are also frequently observed in cancer and are closely associated with disease stage and progression [120]. However, unlike programmed WGD, changes in cell size in cancer cells are not necessarily linked to, or dependent on, changes in DNA content. For example, in DLD1 colorectal cancer cells that have undergone WGD, the resulting 4N cells display significant heterogeneity in terms of cell size, and size does not always correlate with DNA content [ 121, 122] . These findings suggest the existence of alternative regulatory mechanisms that modulate the size of cancer cells.

### Indirect influence on nuclear size

Ploidy changes resulting from WGD are typically accompanied by alterations in cell size. Studies have shown that the relationship between ploidy level and cell size can be modeled via power laws, with scaling exponents typically ranging from 0.7 to 0.9 [119]. These physical changes impose new adaptive challenges on the structure and function of organelles.

A nearly universal correlation exists between nuclear size and cell size ( Figure 1E). Within a given species, larger cells tend to have larger nuclei, and nuclear size usually scales linearly with cell size [ 123, 124] . Although multiple experiments have demonstrated that DNA content and ploidy do not directly determine nuclear size [ 125, 126] , the increase in cell size associated with increased ploidy is generally accompanied by an enlarged nucleus [ 124, 125, 127] .

### Effects on gene expression

As an important model for studying cellular functional diversity and adaptability, WGD in the mammalian liver provides valuable insights into the relationship between gene dosage alterations and transcriptional regulation ( Figure 1F). A microarray analysis of gene expression patterns in mouse hepatocytes with different ploidy levels indicated that ploidy exerts minimal influence on global gene expression. Among the 2N and 4N hepatocytes, only 50 genes were differentially expressed, all with fold changes less than two. Among 4N and 8N cells, only four genes (Gas2, Igfbp2, Nr1i3, and Ccne2) are differentially expressed [ 128, 129] , suggesting that polyploidization in hepatocytes is a stable process not accompanied by the aberrant gene expression typically observed in cancer cells.

Although WGD does not result in global linear amplification of gene expression in hepatocytes, it can selectively enhance tissue-specific functions. The liver is responsible for diverse physiological functions, including nutrient metabolism, immune response, xenobiotic detoxification, and maintenance of metabolic homeostasis. Several studies have shown that, compared with diploid hepatocytes, polyploid hepatocytes exhibit the upregulation of genes involved in nitrogen metabolism, plasma protein synthesis, redox homeostasis, xenobiotic metabolism, and immune responses, with the most notable upregulation observed in immune-related genes [ 130, 131] . A similar phenomenon has been reported in the heart. Since most cardiac energy is devoted to contraction, polyploid cardiomyocytes show significantly elevated expression of genes related to myocardial contraction, with Gene Ontology (GO) enrichment analysis revealing stronger enrichment for upregulated genes than for downregulated genes. These findings suggest that polyploidy can selectively enhance pathways associated with specialized tissue functions.

### Polyploidy enhances stress resistance

Multiple lines of evidence from polyploid organisms to polyploid cells within tissues and organs indicate that WGD plays a key role in enhancing stress resistance ( Figure 1G). One mechanism involves genomic redundancy, which buffers against the deleterious effects of loss-of-function mutations, thereby protecting polyploid cells from harmful genetic alterations [132]. For example, early liver tumor lesions are often characterized by an increased proportion of diploid cells, suggesting that polyploidization may confer a protective effect [133].

Moreover, polyploid cells can increase survival by modulating the expression of stress response genes. In both the heart and liver, polyploid cells frequently upregulate genes involved in defense against pathogens, DNA damage repair, and the oxidative stress response [130], further supporting the role of WGD in improving stress resistance.

### Impact on chromosome stability

Despite the physiological advantages conferred by WGD, it may also lead to CIN ( Figure 1H). The increased number of centromeres and chromosomes following WGD can induce aneuploidy and promote tumorigenesis. Although polyploid cells often spontaneously lose extra centromeres during continued proliferation and most tetraploid or near-tetraploid cells exhibit normal centromere numbers [ 134– 136] , the absence of functional p53 allows supernumerary centromeres to form improper attachments, resulting in chromosome segregation errors and aneuploidy [ 56, 137] . Additionally, the presence of excessive chromosomes may increase the likelihood of segregation errors during mitosis, causing DNA damage and exacerbating CIN [ 138, 139] .

## Detection Methods

Compared with normal diploid cells, cancer cells frequently exhibit aneuploid or polyploid characteristics. Studies have demonstrated that changes in ploidy are closely associated with cancer development and prognosis [140]. Therefore, accurately detecting ploidy in cancer cells is crucial for understanding tumor evolution, heterogeneity, and therapeutic responses.

### Traditional WGD detection methods

**Figure 2:**
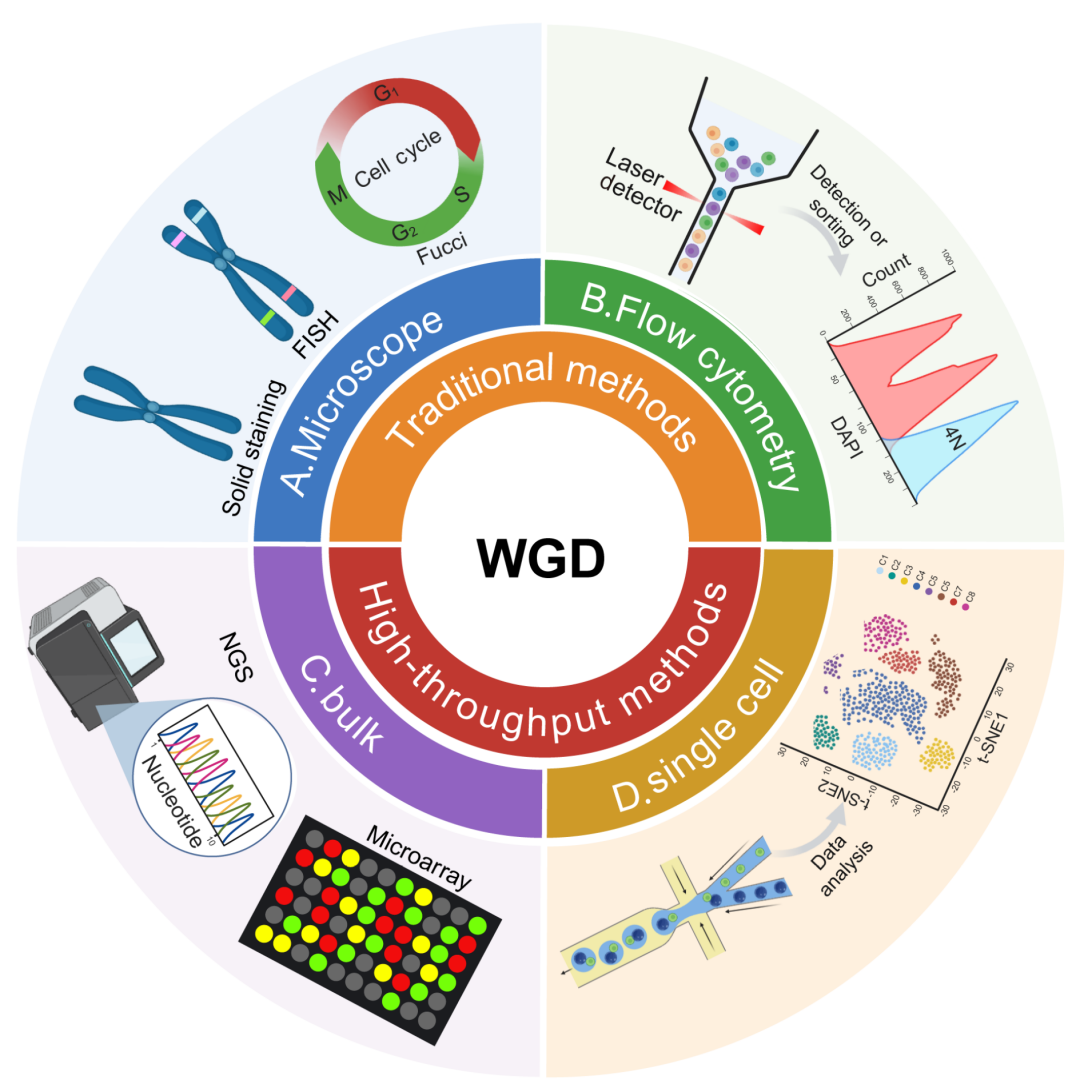
Figure 2 Overview of traditional and high-throughput technologies for detecting WGD Detection strategies for WGD can be broadly categorized into traditional methods (microscopy-based and flow cytometry-based) and high-throughput methods (bulk and single-cell omics), as indicated by the color-coded quadrants. (A) Microscopy-based methods: Conventional karyotyping using solid staining or banding techniques enables direct visualization of chromosome morphology and number. FISH detects specific chromosomal regions in interphase or metaphase cells. The Fucci system labels distinct cell cycle phases using phase-specific fluorescent reporters, allowing identification of G1-phase cells with 4N DNA content, indicative of recent WGD. (B) Flow cytometry: DNA-binding fluorescent dyes are used to measure cellular DNA content, enabling inference of ploidy levels and cell cycle distribution through population-level analysis. (C) Bulk genomic methods: DNA microarrays detect CNVs at sub-chromosomal resolution via hybridization probes. NGS, particularly WGS, allows genome-wide detection of WGD and ploidy abnormalities through read-depth and B-allele frequency analysis. (D) Single-cell approaches: Single-cell DNA or RNA sequencing provides high-resolution detection of WGD and aneuploidy at the individual cell level, revealing tumor heterogeneity and rare subclonal events. Integrated data analysis further enhances sensitivity to polyploidy and subpopulation structure. The figure was created by Biorender.

Traditional WGD detection methods rely primarily on cytogenetic and molecular biology techniques. Conventional karyotyping, which is based on specific chromosomal banding patterns, allows direct observation of chromosomal morphology and numerical variations under a microscope ( Figure 2A). Although it is regarded as the “gold standard” for ploidy detection, this method is limited by low throughput, time-consuming procedures, and the requirement for metaphase chromosome preparation, making it unsuitable for high-throughput studies and complex sample analysis [141]. Flow cytometry is another widely used technique that measures the cellular DNA content by employing dyes that bind to DNA in a stoichiometric manner. It enables the determination of DNA content, cell cycle distribution and ploidy status. However, this technique requires ploidy controls and many cells as input material, and it may introduce bias due to pre-selection of cells on the basis of prior ploidy profiles [142]. In addition, fluorescence in situ hybridization (FISH), a major molecular cytogenetic technique, uses fluorescently labeled DNA probes to hybridize with metaphase chromosomes or interphase nuclei, allowing the detection of specific genes or chromosomal regions. A key advantage of FISH in clinical practice is its ability to analyze chromosomal changes in non-dividing cells directly from cytological or tissue preparations. Variants such as multiplex FISH (M-FISH), spectral karyotyping (SKY), and comparative genomic hybridization (CGH) have simplified the interpretation of complex and unstable cancer karyotypes and are widely applied in both research and clinical cancer diagnostics [ 143– 145] .

The Fluorescent Ubiquitination-based Cell Cycle Indicator (Fucci) system enables real-time visualization of cell cycle phases via fluorescent protein (FP)-tagged degradation domains of cell cycle regulators in living cells. Specifically, G1-phase cells are labeled with Cdt1-fused fluorophores (typically red), whereas S/G2/M-phase cells are marked with Geminin-fused fluorophores (typically green), reflecting their distinct ubiquitin-mediated degradation dynamics. The cells that transition from the G1 phase to the S/G2 phase often appear yellow because the fluorophores overlap [ 146, 147] . A modified version, Fucci (CA), allows clear distinction of S- to G2/M-phase cells [148]. A G1-phase cell identified by FUCCI but with a DNA content of 4N (as typically quantified by DNA dyes) is highly indicative of a recent WGD event [148]. Combined with FACS ( Figure 2B), specific cell populations, such as WGD cells, can be precisely isolated on the basis of their Fucci fluorescence profiles and DNA content [68]. Furthermore, to elucidate the mechanism of WGD generation, histone H2B fused to a fluorescent protein (FP-H2B) is commonly employed to identify mitotic cells. Analysis of WGD cells revealed an absence of typical chromosome condensation (characteristic of endoreplication) or disrupted mitosis (indicative of mitotic slippage). Instead, these cells proceed directly to the next G1 phase, a transition clearly marked by the Fucci reporters [62]. Critically, Fucci technology, combined with long-term live-cell imaging, enables real-time tracking of WGD events at single-cell resolution.

### High-throughput WGD detection methods

In recent years, the rapid development of high-throughput sequencing, single-cell technologies, and machine learning has significantly refined our understanding of WGD in cancer and other complex diseases. These technological developments have enabled highly sensitive and specific detection of ploidy alterations, including genome-wide copy number variations (CNVs) at the chromosomal and subchromosomal levels, thereby facilitating the inference of WGD and related large-scale genomic events. In parallel, emerging computational frameworks, ranging from traditional machine learning to deep learning, are expanding the landscape of model-based ploidy inference and cancer prognosis prediction. However, extracting reliable ploidy signals under constraints such as low sequencing depth and biological noise remains a key technical challenge.

The evolution of genomic technologies, including DNA microarrays, has opened new avenues for profiling chromosomal alterations in tumor cells ( Figure 2C). Compared with conventional metaphase karyotyping, microarrays offer higher resolution for CNV detection at subchromosomal scales [149], although throughput and sensitivity to sample quality remain limiting factors. In contrast, next-generation sequencing (NGS), particularly WGS, has emerged as a cornerstone of cancer genome analysis, offering comprehensive and cost-effective detection of WGD and ploidy abnormalities [150]. By utilizing the differences in DNA content between diploid and polyploid/aneuploid cells reflected in sequencing coverage, tools such as CNAnorm have been developed for tumor purity and ploidy estimation from standard WGS data through genome binning and read depth analysis, with corrections for systematic biases such as GC content and mappability [ 150, 151] . Moreover, because changes in ploidy and purity also affect the distribution of allele frequencies across genomic segments, integrative analyses of sequencing coverage and allele frequencies have become the mainstream framework for CNV and ploidy inference [152]. Tools such as ASCAT [153], absCNAseq [154], PyLOH [155], TITAN [156], Sequenza [157], Accucopy [158], FACETS [159], ABSOLUTE [160], and PURPLE [161] leverage WGS data to jointly estimate tumor purity, ploidy status, and copy number alterations. For instance, in a study conducted by Neil J. Ganem’s research group at Boston University School of Medicine, ABSOLUTE was used to systematically assess WGD status across nearly 10,000 primary tumor samples from The Cancer Genome Atlas (TCGA). This study revealed that WGD-positive tumors present a greater total mutation burden than WGD-negative tumors do and that WGD-positive tumors present distinct patterns of genetic vulnerability.

Despite the strengths of bulk sequencing, its resolution is inherently limited by the averaging of signals across heterogeneous cell populations. Single-cell genome sequencing has overcome this limitation, enabling the identification of ploidy diversity and low-abundance subclones within tumors ( Figure 2D) [ 162– 164] . Current tools commonly used for analyzing single-cell CNVs and ploidy states include SeCNV [165], rcCAE [166], HMMcopy [167], Ginkgo [168], AneuFinder [169], SCOPE [170] and scAbsolute [171]. These tools typically start by partitioning the genome into windows and calculating sequencing coverage for each window. The raw read depths are then corrected and normalized, followed by the application of segmentation algorithms [172] to identify CNV regions. Finally, by integrating signals across entire chromosomes or large genomic segments, the ploidy state of each cell can be inferred. In addition, scPloidy employs an expectation-maximization algorithm to estimate ploidy from scATAC-seq data [173]. Tools such as Numbat [174] and SCEVAN [175] can distinguish tumors from normal cells via single-cell transcriptomes, infer tumor subclones, and identify euploid non-malignant cells [176]. These capabilities are crucial for characterizing tumor heterogeneity, understanding tumor progression, and uncovering mechanisms of therapeutic resistance.

Machine learning and deep learning have further augmented ploidy detection. For example, a Model-Agnostic Meta-Learning (MAML) framework combined with deep neural networks has been adapted for WGD classification in histopathological images across multiple tumor types [177]. These advances may enable rapid, automated prediction of ploidy states in clinical diagnostics.

Notably, ploidy alterations, particularly WGD, usually do not occur in isolation. They are often accompanied by transcriptomic, proteomic, post-translational, and epigenetic changes. Multiomics integration is thus essential for mapping ploidy-related alterations comprehensively. Chang *et al*. [178] integrated genomic, transcriptomic, proteomic, and phosphoproteomic data from the Clinical Proteomic Tumor Analysis Consortium (CPTAC) to systematically characterize WGD events across ten cancer types. Their study revealed tumor type-specific mutational patterns in WGD-positive samples, accompanied by the activation of transcription factors ( *e.g*., E2F and BPTF), increased kinase activity ( *e.g*., CDK1/2 and PAK4), and druggable vulnerabilities, providing insights into precision oncology strategies targeting WGD-driven mechanisms.

In summary, recent technological advancements have significantly enhanced our ability to study WGD and its role in tumorigenesis, progression, and therapeutic targeting. As sequencing and computational methods continue to improve, particularly in extracting reliable signals under challenging conditions, researchers and clinicians are gaining increasingly detailed insights into how genome doubling shapes the molecular evolution of cancer.

## Perspectives

Although significant progress has been made in elucidating the prevalence, mechanisms, and consequences of WGD, many key questions remain. How WGD is precisely regulated across different tissues, how cells selectively tolerate or eliminate polyploid states, and how WGD intersects with immune surveillance and metabolic rewiring are all areas of active investigation. Technological advances such as high-throughput single-cell sequencing, spatial omics, and live-cell imaging are expected to further refine our understanding of WGD dynamics in both physiological and pathological contexts. Moreover, integrating multi-modal datasets through artificial intelligence may uncover previously unrecognized biomarkers or vulnerabilities associated with polyploidy, particularly in cancer. Ultimately, deciphering the dual nature of WGD, as both an adaptive and destabilizing force, will be essential for harnessing its potential in regenerative medicine and for developing ploidy-informed diagnostic and therapeutic strategies.
